# An update on obesity research pattern among adults in Malaysia: a scoping review

**DOI:** 10.1186/s12905-018-0590-4

**Published:** 2018-07-19

**Authors:** Noor Safiza Mohamad Nor, Rashidah Ambak, Norazian Mohd Zaki, Nur Shahida Abdul Aziz, Siew Man Cheong, Mohamad Aznuddin Abd Razak, Muslimah Yusof, Mohamad Hasnan Ahmad, Azli Baharuddin, Megat Rusydi Megat Radzi, Wan Nur Khairunnisa Wan Kozil, Intan Hafizah Ishak, Tahir Aris

**Affiliations:** Institute for Public Health, National Institutes for Health, Ministry of Health Malaysia, Kuala Lumpur, Malaysia

**Keywords:** Scoping review, Obesity, Research pattern, Overweight, Adult, Malaysia

## Abstract

**Background:**

Obesity is a global health burden in the non-communicable diseases and much efforts have been implemented in the past decade in response to the rise of obesity prevalence among the Malaysian population. These include the development of the national policies, health programmes and research activities. The main aim of the scoping review was to identify obesity research pattern among adults in Malaysia in terms of the scopes, topics and the research designs.

**Methods:**

The scoping review was conducted based on the framework by Arksey and O’Malley. The Preferred Reporting Items for Systematic Reviews and Meta Analysis (PRISMA) diagram was used as a guide to record the review process. Articles from year 2008 until 2017 on overweight and obesity among adults aged 18 years and above were retrieved based on the keywords using electronic databases (Embase/Ovid, Pubmed, Cochrane library and Google Scholar). Local journals, Nutrition Research in Malaysia Biblography (2011 and 2016), online local theses databases, virtual library databases were also included in the searches. Consultations with relevant key informants from the National Institutes of Health and local universities were also conducted. Search activities were managed using Endnote software and MS Excelsheet.

**Results:**

The characteristics of the results were described based on the objectives of the review. A total of 2004 articles and reports were retrieved, and 188 articles related to obesity in Malaysia were included in the final review. Scopes and topics of obesity research based on the Nutrition Research Priorities in Malaysia (NRPM) for 11th Malaysia Plan were obesity prevalence, weight loss intervention, association of physical activities and dietary factors with obesity. The majority of obesity research among adults in Malaysia was cross sectional studies and only a small number of intervention studies, qualitative studies and systematic review were indentified. Research gaps were identified in order to make useful recommendations to the stakeholders.

**Conclusions:**

In the past decade, there has been an emerging evidence on obesity research among adults in Malaysia. More obesity research needs to be conducted particularly on obesity intervention among specific gender, qualitative studies, economic cost and genetic factors of obesity.

**Electronic supplementary material:**

The online version of this article (10.1186/s12905-018-0590-4) contains supplementary material, which is available to authorized users.

## Background

Obesity is a global problem in both developing and developed countries, and has become a leading health burden in the Non-Communicable Diseases (NCD). In the past five years, the prevalence of obesity among adults in Malaysia showed a continuing increase of the problem, although a slower increament rate has been reported by the National Health and Morbidity Survey Malaysia (NHMS) 2011 and 2015 [1, 2]. The NHMS reported the prevalence of overweight among adult in Malaysia was 29.4% (NHMS 2011) and 30.0% (NHMS 2015), while obesity prevalence was 15.1% and 17.7% respectively [1, 2]. In response to the rise of obesity problem in Malaysia, various efforts and strategies have been implemented in the past decade to combat this problem. These include a new national nutrition policy and strategies, dietary guidelines, healthy lifestyle campaigns and the development of the Nutrition Research Priorities in Malaysia.

In 2014, the Institute for Public Health conducted a dialogue on obesity research to discuss the magnitude of obesity problem and categories of obesity research conducted by various institutions including from the Ministry of Health, the National Institues of Health and local universities [3]. The majority of topics for obesity research among adults in Malaysia were conducted by the researchers and students at local universities and these included risk factors of obesity, disease related with obesity, perception and body image among obese women, obesity metabolic pathway, obesity biomarkers and knowledge, attitude and practice (KAP). Issues and challenges in conducting obesity research were also highlighted in the research dialogue in terms of the research design, sub population and gap between research and practice.

Following the research dialogue activity, the National Research Priority Malaysia (NRPM) for 11th Malaysia Plan was developed in 2016 under the National Coordinating Committee on Food and Nutrition (NCCFN). Prior to this, the NRPM for 10th Malaysia Plan (2011-2015) was used by the researchers and the programme managers to address the research gap in nutrition [4]. In the latest NRPM (2016-2025), the Technical Working Group on Nutrition Research (TWGNR) has identified 14 scopes of overweight and obesity research priority area with the focus to improve understanding on the epidemiolgy of obesity, effectiveness of the intervention, management of obesity and developing new modalities. To date, evidence on obesity research conducted among adults based on the current NRPM framework is still not known. Therefore, the aims of the present review were to identify various topics of obesity research in Malaysia corresponding to the NRPM conceptual framework, and to make future recommendations on potential future research topics and research design in obesity.

## Methods

Scoping review was applied for this topic with the aim to map the profile of obesity research among adults in Malaysia, which will also allows researchers to identify potential future topics on systematic review and meta analysis in obesity. Scoping review has a similar process as systematic review in identifying the literature or evidence, but scoping review seeks to map the evidence comprehensively rather than to analyse the specific outcomes based on specific questions as in the systematic review. In the present review, adults were defined as respondents aged 18 years old and above, which also include the elderly population. Obesity research was defined as any types of study related to the scope and area of overweight and obesity problems. The conduct of the scoping review utilised an established scoping review framework by Arksey and O’ Malley [5]. The systematic approach to searching, screening and reporting process of the scoping review was enhanced using the current recommendations by Levac, Colqohoun and O’ Brien [6]. Six stages in the scoping review framework were applied, which included (1) identifying the research question, (2) identifying relevant studies, (3) study selection, (4) charting the data, (5) collating, summarising and reporting the results and (6) consultation with the stakeholders and experts in obesity [5].

### Identifying the research question

The present scoping review sought to answer the following research questions:

i. What are the characteristics of obesity research among adult in Malaysia in the past 10 years ago in terms of:

- the number of research conducted in Malaysia
- research design and methodology (qualitative and quantitative studies)
- scope and topics of overweight and obesity research according to the purpose and scope of the NRPM for 11^th^ Malaysia Plan,

ii. What are the research gap in obesity research in Malaysia and what are the future potential research in obesity?

**Fig. 1:**
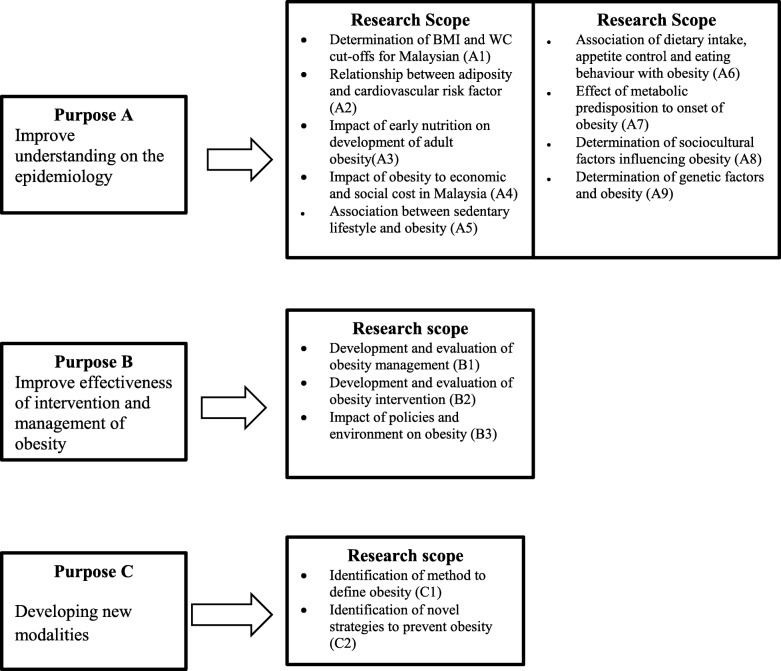
Conceptual framework on obesity research priorities in Malaysia for 11^th^ Malaysia Plan (2016-2025)

The conceptual framework on the purpose and scope for obesity research priority area in the NRPM for 11^th^ Malaysia Plan was used to guide the researchers in the review process and data charting (Fig. 1). Fourteen scopes in the NRPM 2016 were used to map the evidence according to the topics listed in each research scope.

### Identifying relevant studies

A comprehensive search to identify primary studies, reviews, grey literature (including technical reports) on obesity from January 2008 to December 2017 was performed using different resources. These included different electronic databases (Embase, Ovid, Pubmed, Cochrane Library, Google Scholar). Manual searches of the local journals and journal supplements related to obesity studies in Malaysia, Malaysia Nutrition Research Biblography (1985-2010 and 2011-2014), online local theses databases were conducted to maximise the search activities. The library databases and manual searching for dissertations/theses at local universities, relevant websites (World Health Organisations, Ministry of Health Malaysia Virtual Library, Malaysian Research Institute of Ageing) were also used to identify relevant studies. Subject headings, list of keywords and synonyms (obesity, overweight, obesity research, adults, Malaysia) were developed as search terms by the research team members, in order to capture potential studies in the resources (see Additional file 1). The search strategy was developed based on the search terms by the experienced researcher and the research librarian supported the search activities. Boolean operators (OR, AND, NOT) including adjacencies and truncations were used to combine the keywords and related terms during the literature search.

### Study selection

Inclusion criteria for the search were published articles from 2008 to December 2017 related to obesity research among Malaysians aged 18 years and above (adult and elderly). These included articles from primary studies, technical reports and review articles (systematic review or narrative review). Language limit was applied whereby Malay and English language articles were selected. Selection of articles were performed in two stages. In the first stage, researchers (working in pairs) independently screened the titles and abstracts of all resources based on the inclusion criteria and search terms. The Preferred Reporting Items for Systematic Reviews and Meta Analysis (PRISMA) diagram (2009) was used as a guide to record the review process [7]. Selected titles and abstracts were then screened and checked whether the content potentially answered the review questions. Irrelevant abstracts were excluded and the researchers then retrieved full articles of the selected abstracts.

In the second stage, full articles were screened to identify items related to the objectives of the review. Similar to the first stage, each pair (2 researchers) independently reviewed the full articles if they meet the objectives of the review. Data from both researchers were also compared to ensure the consistency of the review and any discrepencies between the reviewers were discussed. Articles were excluded if they are not relevant and did not describe the characteristics of obesity research in Malaysia and the objectives of the review. Relevant articles were then assessed in order to the answer the review questions. The results from the search were managed using the Endnote X5 software and extracted data from the full articles were documented in the Microsoft Excel spreadsheet.

### Charting the data

Based on the conceptual framework on the purpose and scope for obesity research priority area in the NRPM 2016, the researchers developed a standard charting table to categorise the research topics according to the main three domains ( stated as purposes in the NRPM 2016) and these included (A) Improve understanding on the epidemiology of obesity; (B) Improve effectiveness of intervention and management of obesity; and (C) Developing new modalities. The charting table was piloted on 50 articles to ensure the standardised process of charting was applied and common understanding between the researchers on the category of the obesity research topics. General and specific information of the studies were included in the charting table such as author(s), year of publication, objectives or aims of the study, study location and settings, study population (male and/or female adult or elderly), study design and sample size. Emerging topics which was not captured in the charting process were compiled and collated into a new domain as ‘other scopes’.

### Collating,summarising and reporting the results

The results of the extracted data were analysed using descriptive statistics (e.g. percentage) to provide summary characteristics of the studies based on the number and types of studies according to the scopes of obesity research. Data were presented using table of findings based on the characteristics of the studies and the NPRM 2016 framework. The quality of articles were not assessed as it is outside the scope of the present scoping review. Several limitations of the studies were also gathered in order to address the research gap and to make useful recommendations for future research in obesity.

### Consultation with programme managers and experts in obesity

We also conducted consultations with relevant key informants from the Obesity Research Dialoge 2014 to provide insights and additional resources on obesity research and the direction of future research in obesity. These included researchers and experts from the local universities, National Institutes of Health (Institute for Public Heath, Institute for Medical Research, Clinical Research Centre, Institute for Behavioural Research, Institute for Health Management & Institute for Health System Research), Nutrition Division MOH, Nursing Division MOH, Allied Health Division MOH, Non-Communicable Disease Division MOH, Chairperson of the Technical Working Group for Nutrition Research (NPANM) MOH and the president of the Malaysian Association for Study of Obesity (MASO). The appraisal of the quality of each article was not included, in line with the guideline of the scoping review conduct [6].

## Results

**Fig. 2:**
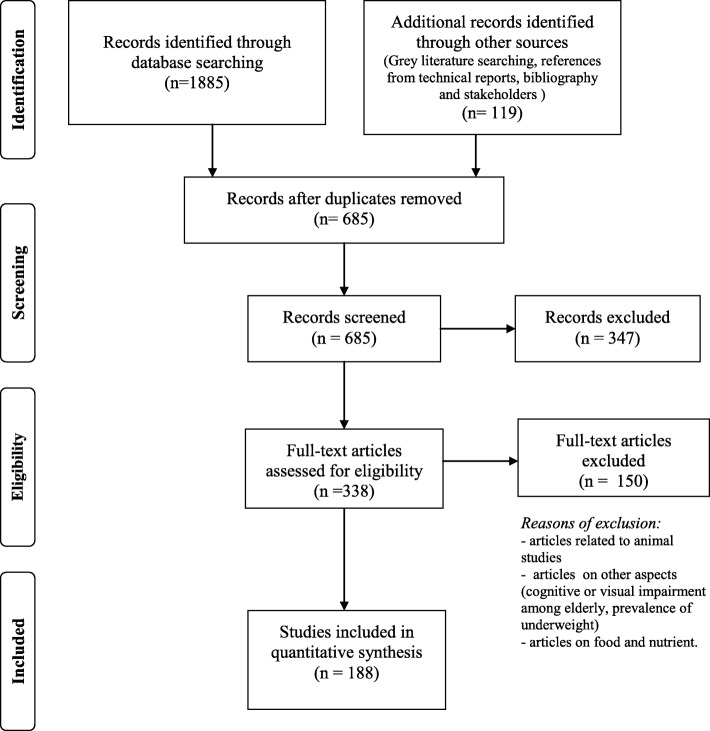
Details of study flow in the different stages of the review

A total of 2004 titles and abstracts were screened at Stage 1 and after screening and removal of the duplicates, 338 potentially relevant full-text papers were included (Fig. 2). 150 full articles were then excluded due to several reasons. These include articles not related to human studies, studies were conducted among adolescent population, studies focusing on other aspects such as cognitive or visual impairment among elderly, prevalence of underweight, dietary intake and studies on food and nutrient components. Following this, 188 documents were included in the charting process and the characteristics of the studies are shown in Table 1. Full list of 188 articles were shown in Additional file 1.

**Table 1:** Study characteristics of obesity research among adult in Malaysia (2008-2017)

Characteristics (*N*=188)		*n* ( %)
**Year of publication**	2008 -2012	93 (49.5%)
	2013-2017	95 (50.5%)
**Sub -population**	Articles among adults	174 (92.6%)
	Articles among elderly	14 (7.4%)
**Conduct of studies**	Articles of studies by MOH researchers	14 (7.4%)
	Articles of studies by non- MOH researchers^a^	174 (92.6%)
**Study design**		
Observational	Cross sectional	148 (78.7%)
	Cohort	1(0.5%)
	Case study	1 (0.5%)
Experimental	Randomised Controlled Trial	2 (1.1%)
	Quasi experimental	1 (0.5%)
	Others (biomarker studies)	23 (12.3%)
Qualitative	Focus group/ In -depth interview	3 (1.6%)
Methodology/Protocol	Obesity intervention/Cohort	4 (2.1%)
Systematic Review/Narrative review		5 (2.7%)
**Sample size (Range)**		50 – 100,000

^a^Non-MOH : local universities, other ministries and organisations other than the Ministry of Health Malaysia

In terms of the number of publications, there was an upward trend of publication in obesity research from year 2011 to 2014 (2011 (*n*=12), 2012 (*n*=29), 2013 (*n*=32), 2014 (*n*=34)), whereby 34 articles related to obesity were published in 2014 and there was a slight decreased trend of the number of publications from year 2015 to December 2017.Sample size of studies ranged from 50–100,000 respondents due to different methodology conducted for particular studies. Table 1 shows that the majority of studies (78.7%) were cross sectional studies and other studies included were cohort, obesity intervention, biomarkers, qualitative studies and systematic review. Almost 93% of studies conducted were among the adults and only 14 articles related to obesity among the elderly population [8–22].

The National Health Morbidity Surveys (NHMS 2011, NHMS 2015) recruited a large scale of respondents around 16,800 adults and reported the prevalence of obesity and overweight among Malaysians adults by age, sex and ethnicity [1, 2]. In the NHMS 2011 and NHMS 2015, the decline of overweight and obesity problems among elderly aged 60 years and above was also reported [1, 2]. Under the umbrella of the NHMS the prevalence of obesity among adult was also reported in the Malaysian Adults Nutrition Survey 2014 (MANS) [23]. Another nationwide study was The Malaysian Cohort (TMC), which reported the prevalence of obesity among multi ethnic urban and rural Malaysians. This prospective study of the non-communicable diseases was initiated in 2005 by the researchers from the National University of Malaysia [24] and more than 100,000 respondents involved in this study. The baseline prevalence of obesity identified from the current cohort was 17.7%.

The majority of obesity research publication in Malaysia were quantitative studies. Out of 188, only three (3) articles on qualitative studies were retrieved (Abdul Aziz et al. [25], Muda et al. [26] and Chang et al. [27]). These studies explored different aspects of obesity problems which include perspectives among women on obesity problems, perceived barriers to weight loss, quality of life and associated factors to reduce weight among overweight and obese homemakers/housewives. Meanwhile, 22 articles related to obesity gene and biomarkers were found to be useful for future direction of obesity research. Majority of these articles were published from a single study among Malay adults in Pahang. The researchers investigated the role of Melanocortin-4 receptor (MC4R), gene variant and resistein levels with obesity [28–49]. The details of several studies and the key findings were highlighted in Table 2.

**Table 2:** Findings of the qualitative studies, elderly studies and biomarkers

No.	Study	Study Design	Sample	Participant Characteristic	Main Findings
1.	Abdul Aziz et al. [2016]	Semi-structured face to face in-depth interview	28	Age: 18 to 59 years old Ethnicity: Malay, Chinese ,Indian Subpopulation: Female , Housewives	• Five main themes associated with obesity problems emerged from the analysis that included ‘personal feelings, beliefs, lifestyles, life issues and effort to reduce weight.
2.	Muda et al. [2013]	Random sampling	421	Age: 20 and above Ethnicity: Malay Subpopulation: Female, Housewives	• Character and behaviour are highly regarded in evaluating a person self worth in the society. • Most respondents were aware of their body weight and indicated an intention to lose weight.
3.	Ching et al. [2009]	Focus group	38	Age: 20 and above Ethnicity: Malay, Iban, Bidayuh. Subpopulation: 21- women 17-Men	• Participants perceived themselves as ugly and felt ashamed of their body size.
4.	Suzana et al. [2012]	Cross sectional study	4746	Age: 60 and above Ethnicity: Malay, Chinese, Indian, others Sub population: Elderly	• Prevalence of overweight and obesity were slightly higher in women (30.3%, 13.8%) compared to men (29.2%. 7.4%) • Malay, Indian ethnicity, higher education level, higher household income, from urban area, and married elderly were predictors of abdominal obesity
5.	Teng et al. [2011]	Randomized controlled study	25	Age: 50-70 years Ethnicity: Malay Subpopulation: Elderly	• Fasting calorie restriction (FCR) group reduced their energy intake for about 18% in 12 weeks time • A significant interaction effect was found in body composition, blood pressure and blood profile in the FCR group • A significant improvement in total DNA rejoining cells and MDA was observed in FCR group
6.	Shahar et al. [2013]	Cross sectional study	160	Age: 60 and above Ethinicity: Malay Subpopulation: elderly in agricultural settlement	• 42.5% of elderly were at risk of malnutrition • Elderly who were at risk of malnutrition have poorer appetite (19.8% of malmutrition risk), decline in functional independent, and more psychosocial problems (depression)
7.	Shahar et al. [2016]	Prospective study	2322	Age: 60 and above Ethinicity: Subpopulation: Elderly in Johor, Selangor, Perak and Kelantan	• The prevalence of successful aging, usual aging and mild cognitive impairment are 11%, 73% and 16%, respectively.
8.	Apalasamy [2014]	Cross sectional study	574	Age: adults Ethnicity: Malay Subpopulation: 464 UM staff and 208 villegars in Bera, Pahang	• ADIPOQ rs17366568 gene was significantly associated with risk of obesity. The frequencies of AG and AA genotypes were significantly higher in the obese group (11%) than in the non-obese group (5%).
9.	Apalasamy [2014]	Cross sectional study	631	Age: adults Ethnicity: Malay Subpopulation: 464 UM staff and 208 villegars in Bera, Pahang	• Resistin levels were not correlated to metabolic parameters such as body weight, waist circumference, body mass index, and lipid parameters. • *RETN* SNPs and haplotypes are of apparent functional importance in the regulation of resistin levels but are not correlated with obesity and related markers.
10.	Chua [2012]	Cross sectional study with convenience sampling	254	Age: 21-80 years Ethnicity: Chinese, Malay, Indian and other ethinicity Subpopulation: adults in Kampar	• Melanocortin receptor 4 (MC4R) V103I gene variant was not associated with obesity

Only five (5) review articles were identified, which inclusive of 3 systematic reviews on obesity and metabolic syndrome [50–52], and 2 narrative reviews [53, 54] and Kambalia [50] described the trend of obesity and overweight among adults in Malaysia from 1996-2009, whereby women have a greater risk for overweight and obesity compared to men. Ng et al. [51] reported a national prevalence of obesity in Malaysia using a systematic analysis for the Global Burden of Disease data. They found that Malaysia has the highest prevalence of overweight and obese compared to other neighbouring countries. Meanwhile, Lim and Cheah [53] reported a review on metabolic syndrome research in Malaysia and the authors stated that there was an emerging evidence on metabolic syndrome among the Malaysian adults. The latest publication by Lim [54] in his narrative review described a comprehensive obesity research profile in Malaysia from 1999 until 2015, comprising of the prevalence of obesity according to age, sex, ethnicity, geographical variations, social and economic factors. The author also highlighted other associated risk factors of obesity, biomarkers of obesity and other diseases (metabolic syndrome, non-communicable diseases, psychiatric disorders, cancer and oral health) [54].

Two articles and 2 technical reports on the methodology and protocol related to obesity research were identified in the present review, which include the methodology of the intervention study (My Body is Fit and Fabulous at Home - MyBFF@home), the population-based longitudinal study for healthy longevity (TUA) for older adults and the dietary intervention protocol of the MyBFF@home [55–58].

**Fig. 3:**
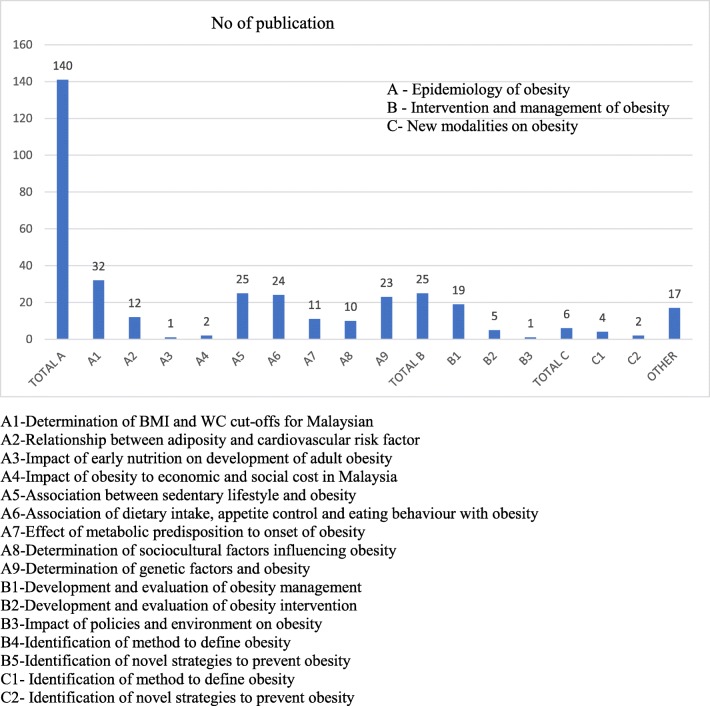
Number of publication on obesity research among adults in Malaysia based on the NRPM for 11^th^ Malaysia plan (*N*=188)

The charting process was then continued based on 188 articles and reports using the NPRM 2016 framework table (Fig. 3). Based on the 3 domains of obesity research, it was found that the majority of research associated with the Domain A – Epidemiology of obesity (*n*=140), followed by Domain B- Intervention and management of obesity (*n*=25) and Domain C- New modalities on obesity (*n*=6). There are several articles were classified as other categories (*n*=17)

## Discussion

Research is an important aspect of the health care system to seek answers for problems, as well as to increase the knowledge and understanding of an area or subject. The findings of the present review has several impacts on the research practice and policy. Firstly, it appears that there was an emerging trend in Malaysia on publication of obesity research in the past 10 years. Studies were conducted at different settings which include the community settings, hospital, universities and army quarters, whereby the majority of studies (more than 80%) were conducted by the researchers and the students at local universities. However, based on the NRPM framework 2016, the majority of the research conducted are related to the understanding the obesity epidemiology domain and only 22% of the publications relevant to the development of the new modalities and effectiveness of the intervention. Therefore, more research funding and support are needed to enhance the research activities related to these domains.

Secondly, researchers in Malaysia are likely to be more active in conducting cross sectional studies compared to other study designs such as RCT, case control, qualitative study including systematic reviews. It was noted that longitunidal studies reporting overweight and obesity among adults and elderly are also still very limited. Although longitudinal studies may require a bigger research fund and human resource compare to other types of study, longitundinal study is useful to set a good platform for researchers to explore various determinants and predictors related to obesity [59]. In addition, the focus of obesity research among the researchers in the MOH is more on the population-based study and one of the the studies is the NHMS, which involves a large scale of respondents in Malaysia. Since 2011, the NHMS was conducted on a yearly basis by the Institute for Public Health, MOH with different focus or health theme, whereby under the umbrella of the NHMS the Malaysian Adult Nutrition Survey (MANS) was also conducted in 2014 to focus on the nutritional status and dietary intake of the adults population in Malaysia. The obesity prevalence was captured in the Nutritional Status Module and all findings were used to support the policy makers and health programme managers in Malaysia. However, only one community- based obesity intervention study (MyBFF@home) was conducted by the MOH researchers from the Institute for Public Health. The majority of the respondents of the MyBFF@home were Malay housewives living in the low cost flats and further research on other ethnic groups are essential to evaluate the effectiveness of the weight loss intervention. There is also a need for researchers in Malaysia to focus on obesity intervention studies among different sub population especially in different ethnicity including the elderly group. Under the umbrella of the MyBFF framework, another intervention study (MyBFF@work) was also conducted and the participants were working adults (male and female workers) in the government sectors in Kelantan, Malaysia [60].

Thirdly, the present review also found that in the last 5 years, other types of obesity studies among specific group of population have emerged mainly among the indigenous group, army, university students, office workers, outpatients at the hospitals or clinics and schizophrenia patients. Despite this emerging trend, obesity research by specific gender in Malaysia is still very limited. According to Kanter and Caballero [61], global gender disparities between obese male and obese female occurred. In the developing countries, change in occupation has decreased the physical activity level among women compared to men, and underemployment may be associated to obesity problems among women [61]. Therefore research to investigate possible socioculturals factors including the occupation status among women in Malaysia are needed to support the obesity intervention programme and the national policies. Articles on narrative and systematic review have also emerged and these types of articles provide a useful insight on obesity trend in Malaysia including the prevalence of metabolic syndrome among the adult population. Based on the NRPM framework, a small number of specific research areas was identified in the past 10 years related to obesity in early life, obesity policy and novel research in obesity. These include articles on obesity intervention, perspectives on obesity, metabolic syndrome and obesity biomarkers. In the charting process, no articles were found on the impact of policies and environment on obesity, and also the economic and social cost of obesity in Malaysia. Therefore, research in these specific areas is important to be conducted in the near future. The economic cost and the health care cost of obesity is substantial and according to Wang and Brownell (2005), the indirects costs caused by the obesity problems in the United States contributed to 10% of lost in the work productivity [62]. Similar aspects should be explored by the researchers in Malaysia in order to assess the economic and social cost of obesity among the adults population.

Lastly, there were some challenges and limitations of this scoping review. The current scoping review only report the characteristics of obesity research by the number of publications but not the actual number of studies conducted by the researchers. For example, some authors published several articles based on the same study. In the present scoping review we have identified 8 articles on biomarkers from only 2 studies. This shows that although there are several publications on obesity biomarkers, the actual study conducted in Malaysia is still very limited. There were also several articles which are categorised as ‘others’, whereby these studies are not included as part of the research NRPM framework. The research team members have worked closely in the categorisation process of the topics to ensure the accurate information was gathered and included in the particular domain. The depth of the literature relevant to the context of the current review was also extensive and requires an expert in research methodology. To ensure the standardised process of data extraction, research team members with diverse experience in different areas contributed to different levels in the screening and selection process of the articles.

## Conclusions

In the light of well established nutrition research framework for obesity in Malaysia, various research topics and publications on obesity in Malaysia have been identified over the past decade. However, focused of the studies in the last 10 years were more on the epidemiology of obesity rather than other categories of obesity research. More research funding is needed to support other research categories such as intervention study, obesity biomarker, cohort study and RCT. The present scoping review was also able to synthesise evidence based on the NRPNM framework. Based on the framework, research is warranted for 2 important research domains on the impact of policies and environment on obesity, and also the economic and social cost of obesity in Malaysia. Findings of this review could support the researchers and the policy makers to make informed decisions about the most appropriate study design on future topics related to obesity.

## Additional file

Additional file 1: Keywords and synonyms for search strategy and Appendix 2 List of articles. (PDF 129 kb)

## Abbreviations

NHMS: National Health and Morbidity Survey
MOH: Ministry of Health Malaysia
MANS: Malaysian Adult Nutrition Survey
NRPM: National Research Priority Malaysia
PRISMA: Preferred Reporting Items for Systematic Reviews and Meta Analysis
TMC: The Malaysian Cohort
RCT: Randomised Controlled Trial
MyBFF@home: My Body is Fit and Fabulous at home
