# Supplementary material for: An update on obesity research pattern among adults in Malaysia: a scoping review
Source: BMC Womens Health. 2018 Jul 19;18(Suppl 1):114. doi: 10.1186/s12905-018-0590-4 (PMC6069511; doi:10.1186/s12905-018-0590-4)
Supplement: Supplementary file 1 — Keywords and synonyms for search strategy and Appendix 2 List of articles. (PDF 129 kb) [file 12905_2018_590_MOESM1_ESM.pdf]

Appendix 1. Keywords and synonyms for search strategy

**Obesity**	**Obesity Research**	**Adult**	**Malaysia**
Overweight<br>Obese<br>Abdominal obesity [MeSH]<br>Morbid obesity [MeSH]<br>Metabolic syndrome [MeSH]<br>Body Mass Index<br>Waist circumference<br>Adiposity<br>Lipid nutrition<br>Cardiovascular risk factors<br>Method definition obesity<br>Metabolic predisposition<br>Onset of obesity<br>Obesity genetic factors<br>Obesity sociocultural factors	Obesity epidemiology<br>Nutritional status<br>Obesity modalities<br>Obesity prevention strategies<br>Overweight and obesity intervention<br>Obesity management<br>Obesity treatment<br>Obesity prevention<br>Obesity evaluation<br>Obesity economic or social impact<br>Obesity policies<br>Cross sectional<br>Clinical trials<br>Population based study<br>Case control obesity<br>Obesity biomarkers<br>Obesity aetiology<br>Development of adult obesity<br>Lifestyle obesity<br>Dietary intake obesity<br>Appetite control<br>Eating behaviour	Wom*n<br>M*n<br>Male<br>Female<br>Adult (s)<br>Working adult(s)<br>Housewive(s)<br>Young adult (s)<br>University student(s)<br>Postgraduate student(s)<br>Office worker (s)<br>Elderly<br>Old people<br>Senior citizen(s)<br>Factory worker (s)	Malaysian population<br>Urban Malaysia<br>Rural Malaysia<br>Perlis<br>Kedah<br>Pahang<br>Kelantan<br>Terengganu<br>Perak<br>Selangor<br>Wilayah Persekutuan<br>Klang Valley<br>Negeri Sembilan<br>Putrajaya<br>Melaka<br>Negeri Sembilan<br>Johor<br>Sabah<br>Sarawak

Appendix 2 List of articles

1. Ainsah O, Razali S, Osman CB, Shah SA. Relationships between antipsychotic medication and anthropometric measurements in patients with schizophrenia attending a psychiatric clinic in Malaysia. 2008; 18:23-7.

2. Ainsah O, Salmi R, Osman C. Binge eating and lifestyle factors in relation to obesity in schizophrenia. Malaysian Journal of Psychiatry. 2008; 17(1).

3. Bee YT, Haresh KK, Rajibans S. Prevalence of Metabolic Syndrome among Malaysians using the International Diabetes Federation, National Cholesterol Education Program and Modified World Health Organization Definitions. Malays J Nutr. 2008; 14(1):65-77.

4. Institute for Public Health: The Third National Health and Morbidity Survey (NHMS III) 2006, Nutritional Status. Kuala Lumpur: Ministry of Health Malaysia 2008.

5. Jamaiyah H, Geeta A, Safiza MN, Wong NF, Kee CC, Ahmad AZ, et al. Reliability and Technical Error of Calf Circumference and Mid-half Arm Span Measurements for Nutritional Status Assessment of Elderly Persons in Malaysia. Malays J Nutr. 2008; 14(2):137-50.

6. Kee CC, Jamaiyah H, Noor Safiza MN, Khor GL, Suzana S, Jamalludin AR, Rahmah R, Ahmad AZ, Ruzita AT, Wong NF et al: Abdominal Obesity in Malaysian Adults: National Health and Morbidity Survey III (NHMS III, 2006). Malays J Nutr. 2008; 14(2):125-35.

7. Moy FM, Atiya AS, Wong ML: Framingham Risk Scores and Anthropometric Measurements in Predicting Cardiovascular Risks among Malay Men. Mal J Nutr 2008, 14(1):57-63.

8. Nazri SM, Imran MK, Ismail IM, Faris AA. Prevalence of overweight and self-reported chronic diseases among residents in Pulau Kundur, Kelantan, Malaysia. Southeast Asian J Trop Med Public Health. 2008; 39(1):162-7.

9. Azmi MY, Junidah R, Siti Mariam A, Safiah MY, Fatimah S, Norimah AK, et al. Body Mass Index (BMI) of Adults: Findings of the Malaysian Adult Nutrition Survey (MANS). Malays J Nutr. 2009; 15(2):97-119.

10. Chang CT, Chang KH, Cheah WL. Adults' perceptions of being overweight or obese: a focus group study. Asia Pac J Clin Nutr. 2009; 18(2):257-64.

11. Fui CS. (Student Thesis) Readiness to Changes Among Obese Patients Attending Counselling Diet Session at Klinik Warga PPUKM. Universiti Kebangsaan Malaysia; 2009.

12. Ramadas A, Kandiah M, Zarida H, Gul A, Faizal J. Obesity and risk of colorectal adenomatous polyps: a case-control study in hospital kuala Lumpur. Malays J Nutr. 2009; 15(1).

13. Sidik SM, Rampal L. The prevalence and factors associated with obesity among adult women in Selangor, Malaysia. Asia Pac Fam Med. 2009; 8(1):2.

14. Wan Nudri WD, Wan Abdul Manan WM, Mohamed Rusli A. Body mass index and body fat status of men involved in sports, exercise, and sedentary activites. Malays J Med Sci. 2009; 16(2):21-6.

15. Zaher ZM, Zambari R, Pheng CS, Muruga V, Ng B, Appannah G, et al. Optimal cut-off levels to define obesity: body mass index and waist circumference, and their relationship to cardiovascular disease, dyslipidaemia, hypertension and diabetes in Malaysia. Asia Pac J Clin Nutr. 2009; 18(2):209-16.

16. Akter SFU, Fauzi A, Nordin M, Satwi S, Mohamed A, Aznan M, et al. Prevalence of cardiovascular risk factors in a selected community at Kuantan, Pahang, Malaysia. Int J Med Med Sci. 2010; 2(10):322-8.

17. Aldahoun MYJ. Diet application for gaining and ideal weight. (Doctoral dissertation, Universiti Utara Malaysia); 2010.

18. Boo N, Chia G, Wong L, Chew R, Chong W, Loo R. The prevalence of obesity among clinical students in a Malaysian medical school. Singapore Med J. 2010; 51(2):126.

19. Chien AJ. (Student Thesis) Discomfort and complications related to overweight and obesity among adults population in Kuala Lumpur. Universiti Kebangsaan Malaysia; 2010.

20. Cheng SL. (Student Thesis) The association of Fatty Acid Binding Protein polymorphism with obesity and type 2 diabetes mellitus in Malaysian subjects. Dissertation (M. Med. Sc.). University of Malaya; 2010.

21. Fihil N: (Thesis) Proportion of Overweight and Obesity and Food Intake Pattern in Malay Pregnant Women in Kuala Lumpur. Universiti Kebangsaan Malaysia; 2010.

22. Haemamalar K, Zalilah M, Neng Azhanie A. Nutritional status of orang asli (che wong tribe) adults in krau wildlife reserve, pahang. Malays J Nutr. 2010; 16(1).

23. Khambalia A, Seen L. Trends in overweight and obese adults in Malaysia (1996–2009): a systematic review. Obes Rev. 2010; 11(6):403-12.

24. Lee C, Norimah A, Ismail M. Association of energy intake and macronutrient composition with overweight and obesity in Malay women from Klang Valley. Malays J Nutr. 2010; 16:251-60.

25. Mohamad H, Suzana S, Ibrahim M, Norshafarina S. Relationship between Appetite, Food Intake and Body Composition among Elderly Malays from an Urban Residential Area in Kuala Lumpur, Malaysia. Malays J Nutr. 2010; 16(3).

26. Moy FM, Hoe VC, Tan CPL, Rosmawati M. Cardiovascular risks among shift and non-shift workers in a public medical centre in Kuala Lumpur. Journal of the University of Malaya Medical Centre. 2010; 13(1):45-9.

27. Poh BK, Safiah M, Tahir A, Siti Haslinda N, Siti Norazlin N, Norimah A, et al. Physical Activity Pattern and Energy Expenditure of Malaysian Adults: Findings from the Malaysian Adult Nutrition Survey (MANS). Malays J Nutr. 2010; 16(1).

28. Saadah N. (Student Thesis) Knowledge and practise and weight management among malay women in Kuala Lumpur. Universiti Kebangsaan Malaysia; 2010.

29. Siti Sa'adiah HN. (Student Thesis) Determinants of Wellness and Illness Among Older Malaysians: A Health Promotion Perspective. In.: Institute for Health Behavioral Research; 2010.

30. Thon CC. (Student Thesis) Transtheoretical Model of Change for weight Control in Malaysian Context. Universiti Malaysia Sarawak; 2010.

31. Yaw YH, Kandiah M, Shariff ZM, Mun CY, Hashim Z, Yusof RM, et al: Pattern of weight changes in women with breast cancer. Asian Pac J Cancer Prev. 2010; 11(6):1535-40.

32. Zaki M, Robaayah Z, Chan S, Vadivale M, Lim T. Malaysia Shape of the Nation (MySoN): a primary care based study of abdominal obesity in Malaysia. Med J Malaysia 2010; 65 Suppl A:143-9.

33. Al-Tahami BA, Bee Y-TG, Ismail AAA-S, Rasool AHG. Impaired microvascular endothelial function in relatively young obese humans is associated with altered metabolic and inflammatory markers. Clin Hemorheol Microcirc. 2011; 47(2):87-97.

34. Azhar A, Zulkarnain H, Ziyadi G, Rahman M. A study of relationship between body mass index and short term outcome of isolated coronary artery bypass graft surgery. J Surg Acad. 2011; 1(2):41-8.

35. Chan P, Fan S, Say Y. No association of peptide tyrosine-tyrosine (PYY) gene R72T variant with obesity in the Kampar Health Clinic cohort, Malaysia. Malays J Nutr. 2011; 17(2):201-12.

36. Gan WY MN, Zalilah MS, Hazizi AS. Differences in eating behaviours, dietary intake and body weight status between male and female Malaysian University students. Malays J Nutr. 2011; 17(2):213-28.

37. Institute for Public Health: National Health and Morbidity Survey 2011 (NHMS 2011). Vol. II: Non-Communicable Diseases. Kuala Lumpur: Ministry of Health Malaysia 2011.

38. Kee C, Jamaiyah H, Geeta A, Ali ZA, Safiza M, Suzana S, et al: Sensitivity and specificity of waist circumference as a single screening tool for identification of overweight and obesity among Malaysian adults. Med J Malaysia. 2011; 66(5):462-7.

39. Kuan P, Ho H, Shuhaili M, Siti A, Gudum H. Gender differences in body mass index, body weight perception and weight loss strategies among undergraduates in Universiti Malaysia Sarawak. Malays J Nutr. 2011; 17(1).

40. Loo T, Maniam T, Ainsah O. Psychiatric Morbidity, Personality Profile and Saliva Cortisol Levels in Overweight and Obese Patients Referred to Dietician Clinics in UKMMC. Malaysian Journal of Psychiatry. 2011; 20(1).

41. Mohamud WNW, Musa KI, Khir ASM, Ismail Aa-S, Ismail IS, Kadir KA, et al. Prevalence of overweight and obesity among adult Malaysians: an update. Asia Pac J Clin Nutr. 2011; 20(1):35-41.

42. Siti Affira K, Mohd Nasir MT, Hazizi AS, Kandiah M. Socio-demographic and psychosocial factors associated with physical activity of working woman in Petaling Jaya, Malaysia. Malays J Nutr. 2011; 17(3):315-24.

43. Teng NIMF, Shahar S, Manaf ZA, Das SK, Taha CSC, Ngah WZW. Efficacy of fasting calorie restriction on quality of life among aging men. Physiology & Behavior. 2011; 104(5):1059-64.

44. Moy FM, Bulgiba A. High prevalence of vitamin D insufficiency and its association with obesity and metabolic syndrome among

Malay adults in Kuala Lumpur, Malaysia. BMC Public Health. 2011;11(1):735.

45. Tan AK, Yen ST, Feisul MI. Determinants of body weight status in Malaysia: an ethnic comparison. Int J Public Health. 2012; 57(2):279-88.

46. Alam P. Nutritional status and eating practices among university students in selected universities in Selangor, Malaysia. Asian J Clin Nutr. 2012;4(3):77-87.

47. Apalasamy Y, Ming M, Rampal S, Bulgiba A, Mohamed Z: Genetic association of SNPs in the FTO gene and predisposition to obesity in Malaysian Malays. Brazilian Journal of Medical and Biological Research 2012, 45(12):1119-1126.

48. Apalasamy YD: (Thesis) Obesity study in Malaysian Malays with focus on candidate genes and biomarkers. University of Malaya; 2012.

49. Chang CT, Lee PY, Cheah WL: The prevalence of cardiovascular risk factors in the young and middle-aged rural population in Sarawak, Malaysia. Malays J Med Sci 2012, 19(2):27-34.

50. Choong SS-Y, Balan SN, Chua L-S, Say Y-H: Preference and intake frequency of high sodium foods and dishes and their correlations with anthropometric measurements among Malaysian subjects. Nutrition Research and Practice 2012, 6(3):238-245.

51. Chua H, Fan S, Say Y: Prevalence of melanocortin receptor 4 (MC4R) V103I gene variant and its association with obesity among the Kampar Health Clinic cohort, Perak, Malaysia. Med J Malaysia 2012, 67(2):234-235.

52. Dunn RA, Tan AK, Nayga RM. Obesity inequality in Malaysia: decomposing differences by gender and ethnicity using quantile regression. Ethnicity & Health. 2012; 17(5):493-511.

53. Goonasegaran AR, Nabila F, Shuhada N. Comparison of the effectiveness of body mass index and body fat percentage in defining body composition. Singapore Med J. 2012; 53(6):403-8.

54. Gopalakrishnan S, Ganeshkumar P, Prakash M, Amalraj V. Prevalence of overweight/obesity among the medical students, Malaysia. Med J Malaysia. 2012; 67(4):442-4.

55. Hasnah H, Amin I, Suzana S. Bone health status and lipid profile among post-menopausal malay women in Cheras, Kuala Lumpur. Malays J Nutr. 2012; 18(2).

56. Hassan NZABM: (Student Thesis) Personality Traits and Impulsivity in Association with Obesity Among the Students of Teachers Training Colleges. Universiti Malaysia Sarawak; 2012.

57. Hazizi AS, Aina MB, Mohd NM, Zaitun Y, Hamid JJ, Tabata I. Accelerometer-determined physical activity level among government employees in Penang, Malaysia. Malays J Nutr. 2012; 18(1):57-66.

58. Karandish M, Sharifi A, Latifi S. Comparison of changes in postprandial serum leptin between healthy and type 2 diabetic individuals. Malays J Nutr. 2012; 18(3).

59. Khor G-L. Food availability and the rising obesity prevalence in Malaysia. IeJSME 2012; 6 Suppl 1: S61-8.

60. Lee H, Fan S, Say Y. Prevalence of RsaI Polymorphism in the 5'Untranslated Region (UTR) of Pro-opiomelanocortin (POMC) Gene and its Association with Obesity in the Kampar Health Clinic Cohort, Malaysia. Malays J Med Health Sci. 2012; 8(1):61-8.

61. Lim S, Fan S, Say Y. Plasma total antioxidant capacity (TAC) in obese Malaysian subjects. Malays J Nutr. 2012; 18(3).

62. Lisa Y, Sook HF. Association of the Cocaine-and Amphetamine-Regulated Transcript Prepropeptide Gene (CARTPT) rs2239670 Variant with Obesity among Kampar Health Clinic Patrons, Malaysia. Malays J Med Sci. 2012; 19(1):43.

63. Lua PL, Salihah N, Mazlan N. Nutritional status and health-related quality of life of breast cancer patients on chemotherapy. Malays J Nutr. 2012; 18(2).

64. Mohamud WNW, Ismail Aa-S, Khir ASM, Ismail IS, Musa KI, Kadir KA, et al. Prevalence of metabolic syndrome and its risk factors in adult Malaysians: results of a nationwide survey. Diabetes Res Clin Pract. 2012; 96(1):91-7.

65. Muhammad MAKK, Samsul D, Alam K, Muhammad UK. Comparison of nutritional status of university students of two Asian countries. Nutr Food Sc. 2012; 42(5):332-8.

66. Ngoh HJ SH, Harsa Amylia MS. Development of Demi-span Equations for Predicting Height among the Malaysian Elderly. Malays J Nutr. 2012; 18(2):149-59.

67. Norlelawati A, Kartini A, Ramli M, Norsidah K, Wan Azizi WS, Tariq AR. Obesity in multiracial schizophrenia patients receiving outpatient treatment in a regional tertiary hospital in Malaysia. East Asian Arch Psychiatry. 2012; 22(2):49.

68. Rampal L, Saeedi P, Aminizadeh Bezenjani S, Salmiah M, Norlijah O. Obesity and associated health related factors among university staff in Serdang, Malaysia. Malays J Med Health Sci. 2012; 8(2):23-32.

69. Rozlan N, Majid HAMA, Abas SS, Danis A, Isa KAM. The association of gestational weight gain and the effect on pregnancy outcome defined by BMI group among women delivered in Hospital Kuala Lumpur (HKL), Malaysia: A retrospective study. Asian J Clin Nutr. 2012; 4(4):160-7.

70. Suzana S, Kee C, Jamaludin A, Noor Safiza M, Khor G, Jamaiyah H, et al. The Third National Health and Morbidity Survey: prevalence of obesity, and abdominal obesity among the Malaysian elderly population. Asia Pac J Pub Health. 2012; 24(2):318-29.

71. Ulaganathan V, Kandiah M, Zalilah M, Faizal J, Fijeraid H, Normayah K, et al. Colorectal cancer and its association with the metabolic syndrome: a Malaysian multi-centric case-control study. Asian Pac J Cancer Prev. 2012; 13(8):3873-7.

72. Wan Abdul M, Firdaus N, Safiah M, Haslinda S, Poh BK, Norimah A, et al. Meal patterns of malaysian adults: findings from the Malaysian adults nutrition survey (MANS). Malays J Nutr. 2012; 18(2).

73. Yasin MM, Daher AM, Nasir NM, Ramli AS, Miskan M, Keat NK, et al. 220 Overweight and Obesity in Malaysia: An Epidemiology Survey. J Hypertens. 2012; 30: e67.

74. Yusuf A, Noor MI, Karim NA, Yahaya R. Changes in body mass index (BMI) and body composition in Malaysian Army (MA) personnel following two weeks of strenuous military training. Defence S and T Technical Bulletin. 2012; 5(2):72-83.

75. Apalasamy YD, Ming MF, Rampal S, Bulgiba A, Mohamed Z: Association of melanocortin-4 receptor gene polymorphisms with obesity-related parameters in Malaysian Malays. Ann Hum Biol. 2013; 40(1):102-6.

76. Azuwani A, Noor Khairiah K, Cheong Y, Kok C, Aw N, Nadiah Mhd S, et al. Body Fat Percentage Distribution of an Orang Asli Group (Aborigines) in Cameron Highlands, Malaysia. Malays J Nutr. 2013; 19(2):205-14.

77. Cheong KC, Yusoff AF, Ghazali SM, Lim KH, Selvarajah S, Haniff J, et al. Optimal BMI cut-off values for predicting diabetes, hypertension and hypercholesterolaemia in a multi-ethnic population. Public Health Nutr. 2013; 16(3):453-9.

78. Chey W-W, Fan S-H, Say Y-H. Association of Fat Mass and Obesity-Associated (FTO) Gene rs9939609 Variant with Obesity Among Multi-Ethnic Malaysians in Kampar, Perak. Sains Malaysiana. 2013; 42(3):365-71.

79. Choo B. (Student Thesis) Obesity Among Malaysian College Students. University of Virginia; 2013.

80. Davey T, Allotey P, Reidpath D. Is obesity an ineluctable consequence of development? A case study of Malaysia. Public Health. 2013; 127(12):1057-62.

81. Heng K, Hejar A, Rushdan A, Loh S. Prevalence of metabolic syndrome among staff in a Malaysian public university based on Harmonised, International Diabetes Federation and National Cholesterol Education Program Definitions. Malays J Nutr. 2013; 19(1).

82. Hossain MG, Wee AS, Ashaie M, Kamarul T. Adult anthropometric measures and socio-demographic factors influencing age at menarche of university students in Malaysia. J Biosoc Sci. 2013; 45(5):705-17.

83. Hussin M, Shahar S, Teng NI, Ngah WZ, Das SK. Efficacy of Fasting and Calorie Restriction (FCR) on Mood and Depression among Ageing Men. J Nutr Health Aging. 2013; 17(8):674-80.

84. Ibrahim S, Karim NA, Oon NL, Ngah WZW. Perceived physical activity barriers related to body weight status and sociodemographic factors among Malaysian men in Klang Valley. BMC Public Health. 2013; 13(1):275.

85. Ihab AN, Rohana A, Manan WW, Suriati WW, Zalilah MS, Rusli AM. The coexistence of dual form of malnutrition in a sample of rural Malaysia. Int J Prev Med. 2013; 4(6):690.

86. Jan Mohamed HJB, Mitra AK, Zainuddin LRM, Leng SK, Wan Muda WM. Women are at a higher risk of metabolic syndrome in rural Malaysia. Women & Health. 2013; 53(4):335-48.

87. Koo H, Robert S, Hamid J. Which is a better anthropometric indicator of cardiovascular risk factors in type 2 diabetes mellitus patients? Waist hip ratio or body mass index? Malays J Nutr. 2013; 19(2):163-72.

88. Loy S, Sirajudeen K, Hamid Jan J. Oxidative Stress in Early Life and Later Obesity Development. Malays J Nutr. 2013; 19(3).

89. Lua PL, Wan Dali W, Shahril M. Multimodal Nutrition Education Intervention: A Cluster Randomised Controlled Trial Study on Weight Gain and Physical Activity Pattern Among University Students in Terengganu, Malaysia. Malays J Nutr. 2013; 19(3).

90. Maher CA, Mire E, Harrington DM, Staiano AE, Katzmarzyk PT. The independent and combined associations of physical activity and sedentary behavior with obesity in adults: NHANES 2003-06. Obesity. 2013; 21(12).

91. Mashmoul M, Azlan A, Khaza'ai H, Yusof BNM, Noor SM. Saffron: A natural potent antioxidant as a promising anti-obesity drug. Antioxidants. 2013; 2(4):293-308.

92. Mohammadi S, Sulaiman S, Koon PB, Amani R, Hosseini SM. Association of nutritional status with quality of life in breast cancer survivors. Asian Pac J Cancer Prev. 2013; 14(12):7749-55.

93. Mustafa J, Salleh NM, Isa ZM, Ghazi HF. Overweight problem among primary health care workers in Suburban District of Hulu Langat, Selangor, Malaysia. Pakistan J Nutr. 2013; 12(3):291.

94. Norafidah A, Azmawati M, Norfazilah A. Factors influencing abdominal obesity by waist circumference among normal BMI population. Malays J Public Health Med. 2013; 13(1):37-47.

95. Ramli A. Obesity and habitual physical activity level among staffs working in a Military Hospital in Malacca, Malaysia. Int Med J Malaysia. 2013; 12(1).

96. Ramli A, Henry LJ, Liang YF, Beh JY. Effects of a worksite health programme on the improvement of physical health among overweight and obese civil servants: a pilot study. Malays Journal Med Sci. 2013; 20(5):54.

97. Ruzita AT CC, Ismail MN, Thavaraj S. Evaluation of Effectiveness Projects on Obesity in 2010 Funded by the Malaysian Health Promotion Board). Malays J Health Sci. 2013; 11(1):49-53.

98. Sanip Z, Ariffin FD, Al-Tahami BAM, Sulaiman WAW, Rasool AHG. Obesity indices and metabolic markers are related to hs-CRP and adiponectin levels in overweight and obese females. Obes Res Clin Pract. 2013; 7(4): e315-20.

99. Schaafsma A, Deurenberg P, Calame W, Van Den Heuvel EG, Van Beusekom C, Hautvast J, et al. Design of the South East Asian Nutrition Survey (SEANUTS): a four-country multistage cluster design study. Br J Nutr. 2013; 110(S3): S2-10.

100. Shahar S, Adznam SN, Lee LK, Yusof NA, Salleh M, Mohamed Sakian NI. A nutrition education intervention for anthropometric and biochemical profiles of rural older Malays with metabolic syndrome. Public Health Nurs. 2013; 30(2):140-9.

101. Shyam S, Arshad F, Ghani RA, Wahab NA, Safii NS, Nisak MYB, et al. Low glycaemic index diets improve glucose tolerance and body weight in women with previous history of gestational diabetes: a six months randomized trial. Nutr J. 2013;12(1):68.

102. Sivapathy S, Chang CY, Chai WJ, Ang YK, Yim HS: Assessment of hydration status and body composition of athlete and non-athlete subjects using Bioelectrical Impedance Analysis. J Phys Educ Sport. 2013; 13(2):157.

103. Soon HK, Saad HA, Taib MN, Rahman HA, Mun CY. Effects of combined physical activity and dietary intervention on obesity and metabolic parameters in adults with abdominal obesity. Southeast Asian J Trop Med Public Health. 2013; 44(2):295-308.

104. Suzana S, Boon P, Chan P, Normah C. Malnutrition risk and its association with appetite, functional and psychosocial status among elderly Malays in an agricultural settlement. Malays J Nutr. 2013; 19(1).

105. Teng NIMF, Shahar S, Rajab NF, Manaf ZA, Johari MH, Ngah WZW. Improvement of metabolic parameters in healthy older adult men following a fasting calorie restriction intervention. The Aging Male. 2013; 16(4):177-83.

106. Yee YSS ZY, Chan YM & Norhaizan ME. Association Between Anthropometric Status, Dietary Intake and Physical Activity with Bone Health Status among Premenopausal Chinese Women in the Klang Valley, Malaysia. Malays J Nutr. 2013; 19(3):293-302.

107. Ying LJ. (Student Thesis) Hubungan Antara Kualiti Tidur dan Obesiti Di Kalangan Wanita Melayu Bekerja Di Lembah Klang. Universiti Kebangsaan Malaysia; 2013.

108. Institute for Public Health: Malaysian Adults Nutrition Survey 2014 (MANS). Kuala Lumpur: Ministry of Health Malaysia 2014.

109. Norshafawati AA, Norhaizan ME. Body Mass Index as the Predictor of High Sensitivity C-Reactive Protein: A Risk Marker of Cardiovascular Diseases. Malays J Nutr. 2014; 20(3).

110. Al-Qalah SAAJ, Ghazi HF, Isa ZM, Karim NA. Dietary weight loss practice among government working women who successfully lose weight in Malaysia. Pakistan J Nutr. 2014. 13(8):486.

111. Apalasamy Y, Moy F, Rampal S, Bulgiba A, Mohamed Z. Genetic associations of the INSIG2 rs7566605 polymorphism with obesity-related metabolic traits in Malaysian Malays. Genet Mol Res. 2014; 13(3):4904-10.

112. Apalasamy YD, Rampal S, Salim A, Moy FM, Bulgiba A, Mohamed Z. Association of ADIPOQ gene with obesity and adiponectin levels in Malaysian Malays. Mol Biol Rep. 2014; 41(5):2917-21.

113. Ariffin FD, Ismail A, Sean VTP, Yusoff Z, Awang SA, Rani WRWA, et al. Improved insulin sensitivity, central systolic pressure and inflammatory indicators achieved with minor weight reduction in overweight and obese subjects given education on lifestyle modification. Asian Biomedicine. 2014; 8(2):185-94.

114. Aris MAM, Khattak MMAK, Abdullah Z, Rus RM, Draman S. Adiponectin Correlates in Malaysians: A Comparison of Metabolic Syndrome and Healthy Respondents. Am J Clin Med Res. 2014; 2(6):106-10.

115. Aziz CBA, Omar N, Abdullah WZ, Jalil RA, Nik WSW, Zakaria R. Reduced fibrinogen, fibrinolytic biomarkers, and physical parameters after a weight-loss program in obese subjects. North Am J Med Sci. 2014; 6(8):377.

116. Azmir A, Norfilza M, Norizam S, Anita MN, Zaiton Z. Identification of circulating microRNAs in young men with central obesity. Asian Pac J Trop Dis. 2014; 4(3):236.

117. Boon SLS. Obesity and dining out: An exploration of dietary trends in urban Malaysia. Master Thesis. University of South Florida; 2014.

118. Chee H, Hazizi A, Barakatun Nisak M, Mohd Nasir M. A Randomised Controlled Trial of a Facebook-based Physical Activity Intervention for Government Employees with Metabolic Syndrome. Malays J Nutr. 2014; 20(2).

119. Cheong KC, Ghazali SM, Hock LK, Yusoff AF, Selvarajah S, Haniff J, et al. Optimal waist circumference cut-off values for predicting cardiovascular risk factors in a multi-ethnic Malaysian population. Obes Res Clin Pract. 2014; 8(2): e154-62.

120. Chew WF, Masyita M, Leong PP, Boo NY, Zin T, Choo KB, et al. Prevalence of obesity and its associated risk factors among Chinese adults in a Malaysian suburban village. Singapore Med J. 2014; 55(2):84.

121. Chin KY, Ima-Nirwana S, Mohamed IN, Ahmad F, Ramli ESM, Aminuddin A, et al. Serum osteocalcin is significantly related to indices of obesity and lipid profile in Malaysian men. Int J Med Sci. 2014; 11(2):151.

122. Fan S-H, Say Y-H. Leptin and leptin receptor gene polymorphisms and their association with plasma leptin levels and obesity in a multi-ethnic Malaysian suburban population. J Physiol Anthropol. 2014; 33(1):15.

123. Ibrahim N, Moy FM, Awalludin IAN, Ali Z, Ismail IS. The health-related quality of life among pre-diabetics and its association with body mass index and physical activity in a semi-urban community in Malaysia-a cross sectional study. BMC Public Health. 2014; 14(1):298.

124. Institute for Public Health: National Health and Morbidity Survey 2014: Malaysian Adults Nutrition Survey (MANS). Vol. II: Survey Findings. Kuala Lumpur: Ministry of Health Malaysia; 2014.

125. Lazin M, Lazim M, Rizman M, Zakaria R, Abdul Jalil R, Suriati Wan Nik W, et al. Association of Changes in Body Composition with Changes in Systemic Oxidative Stress Following Weight Loss Program in Obese Adults Attending Obesity Clinic, Hospital Universiti Sains Malaysia. Open Obes J. 2014; 6(1).

126. Lazin M, Md R, Jalil RA, Wan Muda WAM, Nik W, Suriati W, Zakaria R. USM Behavioural Lifestyle Modification Program Reduces Lipid-Based Cardiovascular Risk in Obese Adults: A Pilot Study. Int Med J. 2014; 21(6).

127. Loy SL, Jan Mohamed HJ. Associations between prenatal nicotine exposure, oxidative stress, and postpartum visceral fat. Women & Health. 2014; 54(2):145-60.

128. Mazland MFB: (Thesis) Obesity-Related Factors Among Prison Wardens in Seremban Prison, Negeri Sembilan. Universiti Kebangsaan Malaysia; 2014.

129. Mirinazhad M-M, Farhangi MA, Jahangiri L, Yaghoubi A. Serum adiponectin concentrations in relation to lipid profile, anthropometric variables and insulin resistance in patients with metabolic syndrome. Malays J Nutr. 2014; 20(3):283-9.

130. MZ NA, Suriani I, Sapian AHM, Simmadorai R, Nasir NSAM. Influence of eating behaviours and psychosocial factors on overweight and obesity among medical students in a public university in Malaysia. Int J Public Health Clin Sci. 2014; 1(1):151-9.

131. Neelakantan N, Narayanan M, de Souza RJ, van Dam RM. Effect of fenugreek (Trigonella foenum-graecum L.) intake on glycemia: a meta-analysis of clinical trials. Nutri J. 2014; 13:7-7.

132. Ne'mat NHB: (Student Thesis) Evaluation of Effectiveness of Worksite Intervention for Obesity Among Overweight Staffs in Hospital Tengku Ampuan Afzan, Kuantan. 2014.

133. Ng M, Fleming T, Robinson M, Thomson B, Graetz N, Margono C, et al. Global, regional, and national prevalence of overweight and obesity in children and adults during 1980–2013: a systematic analysis for the Global Burden of Disease Study 2013. The Lancet. 2014; 384(9945):766-81.

134. Radi F, Hasni M. Obesogens as an environmental risk factor for obesity. Malays J Public Health Med. 2014; 14(3):63-70.

135. Rashidah A, Balkish M, Mohd Azahadi O, Nor Azian M, Syafinaz M, Tahir A. Food label reading and understanding among obese adults: a population study in Malaysia. Int J Public Health Res. 2014; 4(2):449-56.

136. Shariff ZM, Sulaiman N, Jalil RA, Yen WC, Yaw YH, Taib MN, Kandiah M, Lin KG. Food insecurity and the metabolic syndrome among women from low income communities in Malaysia. Asia Pacific journal of clinical nutrition. 2014 Jan 21.

137. Robert SD, Ismail AA-S, Wan Rosli WI: Trigonella foenum-graecum seeds lowers postprandial blood glucose in overweight and obese individuals. Journal of Nutrition and Metabolism 2014, 2014.

138. Said SM, Ismail S: Prevalence and factors associated with overweight and obesity among Malaysian post graduate students in a public university. International Journal of Public Health and Clinical Sciences 2014, 1(1):131-140.

139. Satvin K, Sun P, Leong Y: Prevalence of metabolic syndrome and its relation to body composition in chinese elderly. Asian J Gerontol Geriatr 2014, 9:21-26.

140. Siew-Hui F, Siew-Pheng C, Awang B: Insulin Resistance is the Predominant Pathophysiologic Feature of Hyperglycemia in Newly Diagnosed Overweight and Obese Type 2 Diabetes Mellitus in two University Hospitals in Malaysia. Journal of the ASEAN Federation of Endocrine Societies 2014, 26(2):143.

141. Suriani I, Shamsuddin K, Khalib A, Hazizi A, Latifah A: Ramadan fasting and voluntary fasting-potential weight loss and weight maintenance opportunity for overweight and obese Muslims. International Journal of Public Health and Clinical Sciences 2014, 1(2):29-28.

142. Abdollahi A, Talib MA: Sedentary behaviour and social anxiety in obese individuals: the mediating role of body esteem. Psychology, Health & Medicine 2015, 20(2):205-209.

143. Aniza I, Hayati K, Juhaida M, Taufik JA, Badilla II, Khalib L: Obesity related hypertension-gender specific analysis among adults in Tanjung Karang, Selangor, Malaysia. Malaysian Journal of Public Health Medicine 2015, 15(1):41-52.

144. Apalasamy YD, Ming MF, Rampal S, Bulgiba A, Mohamed Z: Gender-dependent association of a β2-adrenergic gene variant with obesity parameters in Malaysian Malays. Asia Pacific Journal of Public Health 2015, 27(2):NP154-NP165.

145. Apalasamy YD, Rampal S, Salim A, Moy FM, Su TT, Majid HA, Bulgiba A, Mohamed Z: Polymorphisms of the resistin gene and their association with obesity and resistin levels in malaysian malays. Biochemical Genetics 2015, 53(4-6):120-131.

146. Chia P-P, Fan S-H, Say Y-H: Screening of peroxisome proliferator-activated receptors (PPARs) α, γ and α gene polymorphisms for obesity and metabolic syndrome association in the multi-ethnic malaysian population. Ethnicity & disease 2015, 25(4):383.

147. Christopher L, Kosai N, Reynu R, Levin K, Taher M, Sutton P, Sukor N, Das S: Effect of exercise on pulmonary function tests in obese Malaysian patients. Clin Ter 2015, 166(3):105-109.

148. Enny E, Abdul M, Ruhaya H: Oral hygiene care and nutritional status among institutionalised elderly in Kedah and Kelantan, Malaysia. Mal J Nutr 2015, 21(2):207-217.

149. Firouzi S, Barakatun-Nisak MY, Azmi KN: Nutritional status, glycemic control and its associated risk factors among a sample of type 2 diabetic individuals, a pilot study. J Res Med Sci 2015, 20(1):40-46.

150. Institute for Public Health: National Health and Morbidity Survey 2015 (NHMS 2015). Vol. II: Non-Communicable Diseases, Risk Factors & Other Health Problems. Kuala Lumpur: Ministry of Health Malaysia; 2015.

151. Ismail S, Shamsuddin K, Latiff KA, Saad HA, Majid LA, Othman FM: Voluntary fasting to control post-Ramadan weight gain among overweight and obese women. Sultan Qaboos University Medical Journal 2015, 15(1):e98.

152. Jan Mohamed HJB, Yap RWK, Loy SL, Norris SA, Biesma R, Aagaard-Hansen J. Prevalence and determinants of overweight, obesity, and type 2 diabetes mellitus in adults in Malaysia. Asia Pac J Public Health. 2015; 27(2):123-35.

153. Khan S, Saub R, Vaithilingam RD, Safii SH, Vethakkan SR, Baharuddin NA. Prevalence of chronic periodontitis in an obese population: a preliminary study. BMC Oral Health. 2015; 15(1):114.

154. Lee KH, Chai VY, Kanachamy SS, Say YH. Association of UCP1 -3826A/G and UCP3 -55C/T gene polymorphisms with obesity and its related traits among multi-ethnic Malaysians. Ethnicity & Disease. 2015; 25(1):65-71.

155. Low HJ TAK. Determinants of Body Weight Status of University Students: Exploratory Evidence from Universiti Sains Malaysia. Malays J Nutr. 2015; 21(3):285-97.

156. Muda WAMW, Kuate D, Jalil RA, Nik WSW, Awang SA. Self-perception and quality of life among overweight and obese rural housewives in Kelantan, Malaysia. Health Qual Life Outcomes. 2015; 13(1):19.

157. Ng HF, Chin KF, Chan K-G, Ngeow YF. The mRNA expression of soluble urokinase plasminogen activator surface receptor in human adipose tissue is positively correlated with body mass index. Genome. 2015; 58(6):315-21.

158. Phipps ME, Chan KK, Naidu R, Mohamad NW, Hoh B-P, Quek K-F, et al. Cardio-metabolic health risks in indigenous populations of Southeast Asia and the influence of urbanization. BMC Public Health. 2015; 15(1):47.

159. Robert SA, Rohana AG, Shah SA, Chinna K, Mohamud WNW, Kamaruddin NA. Improvement in binge eating in non-diabetic obese individuals after 3 months of treatment with liraglutide–A pilot study. Obes Res Clin Pract. 2015; 9(3):301-4.

160. Rosengren A, Teo K, Rangarajan S, Kabali C, Khumalo I, Kutty V, et al. Psychosocial factors and obesity in 17 high-, middle-and low-income countries: The Prospective Urban Rural Epidemiologic study. Int J Obes. 2015; 39(8):1217-23.

161. Salle DDA, Bong Y, Mohamed AM, Shariff AA, Merican AF. Fat to fit–an Asian obesity management program and the Malaysian case study. 2015.

162. Su TT, Amiri M, Mohd Hairi F, Thangiah N, Dahlui M, Majid HA. Body composition indices and predicted cardiovascular disease risk profile among urban dwellers in Malaysia. Biomed Res Int. 2015; 174821(10):29.

163. Suriani I, Shamsuddin K, Abdul Latif K, Saad HA. The effect of the Malaysian Food Guideline guidance on a group of overweight and obese women during Ramadan. Saudi Med J. 2015; 36(1):40-5.

164. Suriani I, Shamsuddin K, Khalib A, Hazizi A, Fadlan M. Barriers to food quantity intake control and healthy eating among overweight and obese working Malay women in public service. Int J Public Health Clin Sci. 2015; 2(3):81-93.

165. Wan Abdul Manan W, Kum C, Lee Y. Body Somatotype, Anthropometric Characteristics and Physical Activity of College-Age Adults in Selected Institutions of Higher Learning in Kelantan, Malaysia. Malays J Nutr. 2015; 21(1).

166. Yang W, Burrows T, MacDonald-Wicks L, Williams L, Collins C, Chee W. Studying the family diet: an investigation into association between diet, lifestyle and weight status in Malaysian families. Malays J Nutr. 2015; 21(2):139-54.

167. Abdul Aziz N, Mohd Zaki N, Mohamad Nor N, Ambak R, Cheong S. Perspective on Obesity Problems and Associated Factors to Reduce Weight among Overweight and Obese Housewives: A Qualitative Study. J Womens Health, Issues Care 5 2016; 6:2.

168. Ahmad Ali Z, Mala AM, Azli B, Rusidah S, Kee CC, Noor Ani A. Prevalence and socio-demographic determinant of overweight and obesity among Malaysian adult. Int J Public Health Res. 2016; 6(1):661-9.

169. Ahmad N, Adam SIM, Nawi AM, Hassan MR, Ghazi HF. Abdominal obesity indicators: waist circumference or waist-to-hip ratio in Malaysian adults population. Int J Prev Med. 2016; 7.

170. Badrasawi M, Suzana S, Zahara A, Devinder K: Nutritional, Physical and Cognitive Status among Pre-Frail and Frail Malaysian Older Adults. Malays J Nutr. 2016; 22(3):351-61.

171. Bibi Nabihah AH, Suzana S, Hasnah H. Association between Isoflavones Consumption and Cognitive Function and Comorbidities among Older Adults Residing in the State of Johor, Malaysia. Malays J Nutr. 2016; 22(1):29-39.

172. Lim K, Cheah W. A Review of Metabolic Syndrome Research in Malaysia. Med J Malaysia 2016; 71 Suppl 1:20-8.

173. Lim KG. A Review of Adult Obesity Research in Malaysia. Med J Malaysia 2016; 71 Suppl 1:1-19.

174. Mohamad Nor N, Ambak R, Omar A, Shahar S, Abdul Aziz NS, Mohd Yusoff MF et al. Methodology of the My Body is Fit and Fabulous at Home (MyBFF@ home): An Intervention Study to

Combat Obesity Among Housewives in Malaysia. J Womens Health, Issues Care. 2016; 5:2.

175. Noor Safiza MN, Rashidah A, Nur Shahida AA, Fazliana M, Cheong SM, Mala M, Yeo PS, Nor Azian M, A.Z L, Ahmad Ali Z et al: Dietary Intervention Protocol for My Body Fit and Fabolous at Home MyBFF@Home: An Intervention Study to Combat Obesity Among Housewives in Klang Valley: Institute for Public Health 2016.

176. Noor Safiza MN, Rashidah A, Nur Shahida AA, Fazliana M, Cheong SM, Mala M, Yeo PS, Nor Azian M, A.Z L, Ahmad Ali Z et al: MyBody is Fit and Fabolous at Home: An Intervention Study to Combat Obesity among Housewives in Klang Valley (MyBFF@Home): Phase 1-Development of the weight Reduction Intervention Package: Institute for Public Health; 2016.

177. Nur FS, Sakinah H, Rosminah M. Prevalence of geriatric malnutrition and its associated factors at the Hospital Universiti Sains Malaysia, Kelantan. Malays J Nutr. 2016; 22(1):41-53.

178. Nurdiana A, Ahmad A, Singh R, Clark B. Assessing physical activity levels of elderly Malays living in semi-rural areas using tri-axial accelerometer. Malays J Nutr 2016; 22(3):363-74.

179. Roszanadia Rusali SS, Lee Xiao Wen, Zahara Abdul Manaf. Effectiveness of a Structured Weight Management Programme at Workplace among Employees of a Petroleum Industry in Malaysia. Jurnal Sains Kesihatan Malaysia (Malays J Health Sci) 2016; 14(2):49-56.

180. Sakinah H SNASS. Determination of Calf Circumference Cut-Off Values for Malaysian Elderly and its Predictive Value in Assessing Risk of Malnutrition. Malays J Nutr. 2016; 22(3):375-87.

181. Shyam S, Fatimah A, Rohana A, Norasyikin A, Nik Shanita S, Chinna K. Effect of Including Glycaemic Index (GI) Nutrition Education, within the Conventional Healthy Dietary Recommendation Framework, on Body Weight and Composition of Women with Prior Gestational Diabetes Mellitus: Results from a One-Year. 2016.

182. Xian TS, Ibrahim N, Johari N, Rusli R, Manaf ZA. Obesity is Associated with More Sick Leave and Lower Quality of Life Among Malay Male Security Officers. Jurnal Sains Kesihatan Malaysia (Malays J Health Sci) 2016; 14(2).

183. Shahar S, Omar A, Vanoh D, Hamid TA, Mukari SZ, Din NC, et al. Approaches in methodology for population-based longitudinal study on neuroprotective model for healthy longevity (TUA) among Malaysian Older Adults. Aging Clin Exp Res. 2016 ;28(6):1089-104.

184. Cheah WL, Chang CT, Hazmi H, Muda W, Manan W. Gender and racial differences in the cardiovascular risk factors among overweight and obese rural adults, Kuching and Samarahan division, Sarawak, Malaysia. J Nutr Metab. 2016;2016.

185. Chan YY, Lim KK, Lim KH, Teh CH, Kee CC, Cheong SM, et al. Physical activity and overweight/obesity among Malaysian adults: findings from the 2015 National Health and morbidity survey (NHMS). BMC Public Health 2017; 17(1):733.

186. Norshariza J, Siti F, Aini Z, Betti S, Neoh M, Aeininhayatey A, et al. Prevalence of malnutrition among hospitalised adult cancer patients at the National Cancer Institute, Putrajaya, Malaysia. Malays J Nutr. 2017; 23(2):161-74.

187. Baharudin A, Ahmad MH, Zaki NA, Cheong KC, Salleh R, Sallehuddin SM, et al. Changes in nutritional status among malaysian adults population from 2003 to 2014. Southeast Asian J Trop Med Public Health. 2017; 48(3):682.

188. Kong JP, Jok L, Ayub AB, Bau RA. Worksite weight management program: A three-months intervention study in a primary health care setting. Nutrition & Food Science. 2017; 47(4):490-510.
